# Impacts of the COVID-19 pandemic on scientists’ productivity in science, technology, engineering, mathematics (STEM), and medicine fields

**DOI:** 10.1057/s41599-022-01466-0

**Published:** 2022-12-03

**Authors:** Seulkee Heo, Alisha Yee Chan, Pedro Diaz Peralta, Lan Jin, Claudia Ribeiro Pereira Nunes, Michelle L. Bell

**Affiliations:** 1 School of the Environment, Yale University, New Haven, CT USA; 2 Department of Chemical and Environmental Engineering, School of Engineering and Applied Science, Yale University, New Haven, CT USA; 3 Administrative Law Department, School of Law, Universidad Complutense de Madrid, Madrid, Spain; 4 School of Public Health, Yale University, New Haven, CT USA; 5 Graduate Program in Law, School of Law, Federal University of Amazon, Manaus, Amazonas Brazil

**Keywords:** Environmental studies, Education

## Abstract

While studies suggested adverse impacts of COVID-19 on scientific outputs and work routines for scientists, more evidence is required to understand detailed obstacles challenging scientists’ work and productivity during the pandemic, including how different people are affected (e.g., by gender). This online survey-based thematic analysis investigated how the pandemic affected scientists’ perception of scientific and academic productivity in the science, technology, engineering, and mathematics (STEM) and medicine fields. The analysis examined if inequitable changes in duties and responsibilities for caregiving for children, family, and/or households exist between scientists who are mothers compared to scientists who are fathers or non-parents. The survey collected data from 2548 survey responses in six languages across 132 countries. Results indicate that many scientists suffered from delays and restrictions on research activities and administrations due to the lockdown of institutions, as well as increased workloads from adapting to online teaching environment. Caregiving responsibility for children and family increased, which compromised time for academic efforts, especially due to the temporary shutdown of social supports. Higher percentages of female parent participants than male parent participants expressed such increased burdens indicating unequal divisions of caregiving between women and men. A range of physical and mental health issues was identified mainly due to overworking and isolation. Despite numerous obstacles, some participants reported advantages during the pandemic including the efficiency of online teaching, increased funding for COVID-related research, application of alternative research methodologies, and fluidity of the workday from not commuting. Findings imply the need for rapid institutional support to aid various academic activities and diminish gender inequity in career development among academicians, highlighting how crisis can exacerbate existing inequalities.

## Introduction

Even though there is not definitive start date of the pandemic and the pandemic began at different times throughout the world, the COVID-19 pandemic, announced by the World Health Organization (WHO) on March 11, 2020 (WHO, 2020), resulted in global mitigation measures such as physical/social distancing and stay-at-home interventions intending to reduce virus transmission. Many research institutions and universities partially shutdown and severely reduced onsite academic activities in many countries (Omary et al., 2020). This significantly changed teaching and research environments for scientists. As universities quickly canceled in-person classes and training programs and instituted online teaching (Tarkar, 2020), substantial challenges were created for students and teachers (Coyne et al., 2020).

The COVID-19 pandemic affected scientists in different fields unevenly (Myers et al., 2020) and the changes in work routines have been especially significant in the science and engineering fields for which research and teaching activities include physical laboratory resources, living animals, and time-sensitive experiments (Mehta et al., 2022). For example, research involving animal subjects was impacted by halts in new animal orders and experiments. Ongoing or new projects requiring human samples or field work were not permitted unless they were relevant to COVID-19 research, and “dry” research that does not require “wet” laboratories was shifted to remote work (Omary et al., 2020). The COVID-19 pandemic has adversely affected clinical trials in the medicine, health care, and public health fields with most trials being delayed or deferred. Ethical issues raised during the pandemic led to the debates in these fields between utilizing the best opportunity to conduct COVID-19 clinical research and drawing efforts toward the new mission of providing clinical care to patients affected by the pandemic (Hashem et al., 2020). Thus, it is anticipated that the institutional-level actions to address COVID-19 brought significant changes in work routines and productivity in these fields that may be different from other research or educational fields.

Several studies explored changes in productivity in academic fields during the pandemic by focusing on publications and the number of hours worked. A survey study for faculty and principal investigators found that total working hours declined for over half of the survey participants by April 2020 in US and European countries, whereas 18% of participants reported increased working hours (Myers et al., 2020). The decreased time devoted to work was largest for scientists in fields involving physical laboratory activities. Although the number of research articles on COVID-19 increased during the early phase of the pandemic (Älgå et al., 2020), the numbers of publications and work hours cannot fully reflect quality or outputs of other scientific work including teaching, grant writing, mentorship, or academic service. Furthermore, although these results provide empirical findings for output of academic work during the pandemic, more information is needed on the various situations that hinder academic activities and scientific productivity. Therefore, more understanding is needed regarding challenges caused by the pandemic both onsite and offsite and how such challenges affect scientists’ productivity and well-being.

Numerous studies focused on disproportionate changes in publications between men and women during the pandemic. The number of COVID-19 articles in medical journals with a female first author was 19% lower than those with a male first author in March–April 2020 (Andersen et al., 2020). Submissions of articles in public health increased overall during January–May 2020 compared to the pre-pandemic period in the US, by 23.8% for men and 7.9% for women (Bell and Fong, 2021). Studies suggest that the pandemic exacerbated existing gender disparities for publications and career progress (Staniscuaski et al., 2021; Breuning et al., 2021). Many studies found that women usually carry a greater burden of domestic responsibilities and childcare (Zamarro and Prados, 2021). The misconception that women do not have time for collaboration due to other duties including childcare (Clancy, 2020) is another obstacle faced by female scientists. Pursuing academic careers while being a mother can be more challenging in male-dominated areas such as science, technology, engineering, and mathematics (STEM)-related fields (Staniscuaski et al., 2021). During the pandemic, increased caregiving responsibility for small children was aggravated due to the closure of childcare facilities and affected gender differences in publications and work hours (Collins et al., 2021; Krukowski et al., 2021). These studies shed light on the need for understanding inequities of productivity by gender and status as a parent for scientists during times of crisis, such as the pandemic. Further, more evidence is needed about how the pandemic affected gender differences in the division of childcare as almost all parents needed to spend more time at home and work remotely during the pandemic (Zamarro and Prados, 2021).

This survey-based qualitative analysis had two specific goals. The first is to identify scientists’ perceptions and experiences regarding their scientific/academic productivity during the COVID-19 pandemic. We focus on work productivity for scientists in terms of research, teaching, and mentoring/supervising, although productivity in academic and scientific fields can be defined more broadly including clinical work, administrative work, outreach, public service, and leadership. While many changes for work environment and conditions were experienced along with competing responsibilities, we aimed to identify and summarize both positive and negative experiences of scientists regarding productivity during the pandemic. Second, we aimed to examine our hypothesis of inequitable changes in duties and responsibilities for caregiving for children, family, and/or households between scientists who are mothers compared to scientists who are fathers or non-parents.

## Methods

### Survey recruitment

This study was approved by the Yale University Institutional Review Board. We conducted a study using an online survey questionnaire and summaries of this survey are presented elsewhere with quantitative analyses related to the mental health and well-being of scientists in STEM, medicine, public health, or other areas of science/engineering (Heo et al., 2022). This paper utilizes participants’ responses to an open-ended question in the questionnaire. The survey study was designed to investigate how the COVID-19 pandemic affects scientists’ work productivity and mental health. This survey targeted scientists working in research and/or educational institutions, government agencies, industry, or other institutions for STEM, medicine, public health, or other areas of science/engineering (hereafter referred to as ‘STEMM fields’). Three screening questions were used to identify participants who satisfied the following criteria and were given the remainder of the survey: (1) age >18 years, (2) scientists in STEMM fields, and (3) not a student.

We recruited participants in two ways. We advertised on social media (Facebook, Instagram), with a brief description and link that directed persons to our questionnaire, starting with the online consent form. We also manually distributed this link to email addresses registered at citation databases such as Scopus and PubMed for articles published 2017–2021 in SCI-level journals of various categories of STEMM fields, using an approach applied in previous research (Deryugina et al., 2021). Persons who received our email were asked to click the survey link for their preferred language and were directed to the survey, starting with the online consent form. The questionnaire was available in some of the world’s most frequently spoken languages (Lane, 2021): English, Mandarin, Spanish, Portuguese, Japanese, and Korean. The survey was open from October 5 through December 31, 2021. Thus, responses represent perceptions of a 2-year period (2020–2021) since the spread of COVID-19, although we recognize that there is not definitive start date for the pandemic and that the pandemic took place at different times throughout the world. We included an open-ended, optional question, “How has COVID-19 affected your overall productivity?”, with a 300 maximum word count. Open-ended responses are widely used to explore participants’ experiences and perspectives in a variety of fields (Feng and Behar-Horenstein, 2019).

### Thematic analysis

Thematic analysis was used to identify patterns of themes in the open-ended survey data to understand scientists’ experiences regarding productivity and work during the pandemic. A thematic analysis approach was chosen as it allows interpretation of large datasets by sorting them into broad themes, which can capture and summarize core points of coherent and meaningful patterns in the responses (Braun and Clarke, 2006; Kiger and Varpio, 2020). These themes recur across participants or datasets and cluster around a central organizing concept (Braun and Clarke, 2006). Themes within the survey responses were identified in an inductive (“bottom-up”) approach because an open-ended question was used rather than structured multiple questions with primarily defined themes. Thus, identified themes were data-driven. We followed the Standards for Reporting Qualitative Research (SRQR) in conducting and reporting the thematic analysis (Supplementary Table S1) (O’Brien et al., 2014).

Responses from the five non-English questionnaires were translated into English using a certified, professional translation service (CQ Fluency). Only non-English responses to the open-ended question were shared with translators, and no other information from the survey data was provided. English and translated non-English responses were combined in a single file. In total, the study included responses from 2548 participants. We iteratively read the raw responses noting initial ideas to draw a combination of inductive themes driven from the data.

After a re-reading process to become familiar with the data, we identified emerging and meaningful patterns (“code”) relevant for changes/difficulties/challenges for work and productivity during the pandemic. We developed and modified the codes as we read through participants’ responses. After finding the meaningful codes, we categorized them into potential themes to build the structure of primary code and lower-area codes. Researcher’s judgment was required to determine what proportion of the data needs to display evidence of the theme for it to be considered a reoccurring theme (Braun and Clarke, 2006). The initially identified codes were regrouped, edited, or removed as the reading progressed. The structured groups of codes were verified by two investigators who performed the iteration of reading through the responses.

After defining the potential codes, we examined how the codes relate to each other. In this stage, researchers re-read survey responses to ascertain whether identified themes and thematic maps represent the dataset. These potential codes became sub-themes and were grouped into several meaningful themes. The developmental process of potential themes and sub-themes are shown in Supplementary Figs. S1–S2. After finalizing relationships between identified themes and sub-themes, we assessed whether the impact of each sub-theme was positive or negative for productivity based on participants’ assessments and responses related to each sub-theme that repeatedly appeared in their responses. A sub-theme could have positive or negative impacts on productivity, or both.

We generated word clouds analyzing the most frequently mentioned 1000 words using Nvivo Ver 12 software. Stacking words in cloud form ensures that the most found words are assigned the largest font size. The visualization using word clouds provides an intuitive way to find major themes and is often used for text documents (Lohmann et al., 2015). This analysis assumes that important and significant words appear in responses more frequently (Carley, 1993). Nvivo’s queries extracted responses mentioning words of five or more letters.

We applied text search analysis to analyze and compare experiences regarding caregiving for children, family, and domestic work between female and male scientists who are also parents. While we recognize the critical importance of studying non-binary genders and the difference between sex and gender (Torgrimson and Minson, 2005; Reisner et al., 2016; Peters and Norton, 2018; Spizzirri et al., 2021), our text search analysis focused on self-identified female and male genders as the percentage of non-binary gender participants was low (0.9%) and previous evidence mainly focused on gender inequality of caregiving between female and male scientists (Zamarro and Prados, 2021). Using the text search query of Nvivo, we compared the number of participants mentioning search words between female and male participants and parent and non-parent participants. Stemmed variants were considered as the same word (i.e., “research” and “researcher”). We searched the following select words relevant for caregiving for children, family, and households: child, childcare, children, son, daughter, baby, kid, elder, daycare, school*, parent*, mother, father, dad, mom, household, chore, and domestic. The asterisk indicates any combination of letters, reflecting stemmed variants as the same word.

## Results

### Characteristics of participants

Our study participants were diverse in terms of position, major, career stage, age, and region. Supplementary Table S2 summarizes participants’ characteristics. About 86% of responses were collected in English. Respondents were 55.8% male and 43.3% female. Approximately 56% were assistant professors, associate professors, or professors. About 41.5% were parents of children age <18 years living with them. About 17.3% of mothers and 15.3% of fathers were the primary caregiver for their young children. About 9.7% of mothers and 19.9% of fathers reported that their spouse/partner was the primary caregiver. The percentage of participants who had help for childcare from other family members, in-house paid childcare providers, or childcare facilities was 7.1% for mothers and 6.8% for fathers. The fraction of responses from North America and European countries were similar (34% each). The region with the next highest percent of responses was Asia, with 16.9%. The field with the highest responses was medicine/public health, accounting for 39.0%, followed by 22.7% for biology. A large portion of participants was in medicine and health care fields, possibly because these fields were particularly affected by the pandemic and such researchers may have been more inclined to complete this survey. About 68.5% percent of the participants were age 35–59 years.

### Results for thematic analysis

**Fig. 1:**
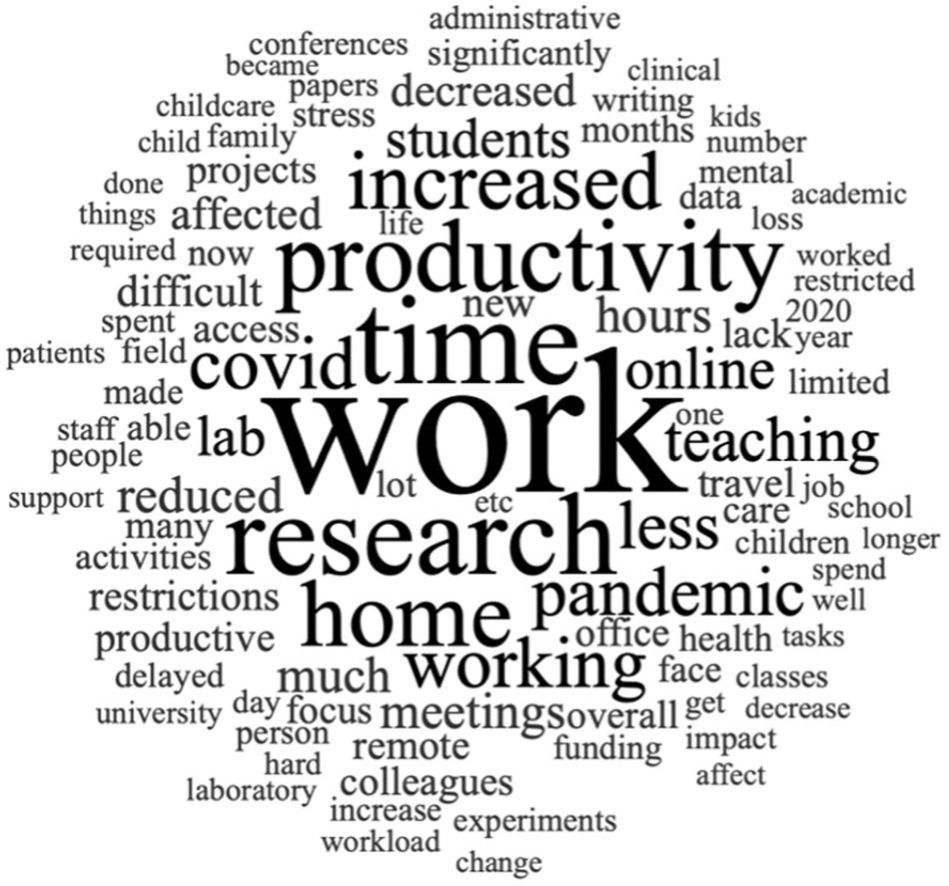
Word Cloud: most frequently mentioned words in the survey responses among the total survey participants. The size of words visualized the frequency of the words found in the survey.

We identified sub-themes regarding the productivity of scientific work under five major themes (Table 1). Initial versions (versions 1 and 2) of potential themes and sub-themes are shown in Supplementary Figs. S1 and S2, and the final version is in Fig. 1. In the following sections, we describe the identified themes and demonstrate the themes by highlighting relevant quotes from participants. Many participants reported both “positive” and “negative” changes during the pandemic regarding their work and productivity. For example, one participant wrote: “In some ways, the productivity declined due to homeschooling kids and having to abandon some data collection. However, I found working from home more balanced in terms of my energy and more focused for writing tasks (participant ID2517).” We note that statements from participants highlighted in the following sections represent many themes that simultaneously influenced to increase or decrease the productivity.

**Table 1:** Summary of themes and sub-themes for changes in work affecting overall productivity among the participants.

Theme	Sub-theme	Positive/negative
For productivity		
Delays and restrictions	Changing COVID regulations	N
	Lack of staff/support	N
	Banned access to lab/office/travel and recruitment	N
	Shortage in equipment	N
	Canceled grants and delayed publication	N
Changes in responsibilities	Teaching burdens and online supervision	P/N
	No or little time for research	N
	Increased burden for child/eldercare and domestic work	N
	Increased number of online meetings	N
Deterioration in health	Physical health	N
	Mental health	N
Insufficient at-home work environment	Blurred boundary between work and life	N
	Insufficient workspace and/or set-up	N
	Lack of in-person interactions	N
Increased flexibility and other positive aspects	Teaching environment	P
	Grants for COVID-19 research	P
	Alternative methods	Somewhat P
	Fluidity of workday	P

*P* = positive aspects, *N* = negative aspects, Somewhat *P* = somewhat positive.

The same changes in work environment, culture, and conditions brought different “voices” regarding productivity and mental health. A notable example is working at home. Some participants described working at home as “distracting” and note “lack of materials for work”, whereas others were “more comfortable”, “efficient”, “more balanced”, or “fluid”, and were “being able to better focus”. Statements regarding the extra workload of online meetings during the pandemic contrasted with other statements referring to flexibility and efficiency of online meetings. The positive aspect of remote working (e.g., fluidity) was contrasted by the negative aspects of lack of face-to-face interactions.

Increases in the number of hours worked did not always mean increased productivity. Work productivity may remain the same or decrease despite increased working hours due to factors including lack of assistance and resources, changing COVID-19 policies, and mental stress of the pandemic. In the quotes, we found it important to distinguish between language about changes in the number of work hours and perception of work productivity. Supplementary Table S3 presents selected de-identified supporting quotes from participants as evidence to support each theme.

#### Theme 1: Delays and restrictions

Changing COVID-19 regulations: The first sub-theme was the delay or shutdown of the work due to newly established and constantly changing COVID-19 policies at institutions. Many participants noted that time spent discussing or establishing COVID-19 policies at institutions consumed much of their time that could have been used for other academic labors.

##### Example response

“Most of the reduction in scientific productivity went into ever-changing administrative means, regulations, communications, etc. to adopt to new pandemic measures (ID1616, Austria).”

#### Lack of staff and support

Lack of efficient administrative assistance contributed to delays in scientific work. Many participants noted lack of staff and support in their institutions during the pandemic. Lack of efficient administrative assistance caused challenges such as decreased time for research, delays in recruitment of researchers/staff, less support for grant applications, decreased information technology (IT) support, and approval of budgets. The identified reasons for the lack of staff included institutional financial challenges and illness of staff and their family, which sometimes resulted in permanent leave of the staff. Many participants mentioned that administrative support significantly decreased or slowed as staff worked from home and were less responsive. Also, staff had more duties for addressing COVID-19-related policies, leaving less time for other work.

##### Example response

*“*The university eliminated many positions so much of administrative work now falls on me (ID519, USA).”

#### Banned access to laboratories/office/travels and recruitment

The ban on access to laboratories, offices, and work travel caused a severe loss of activities and outputs for research and education. Many participants, especially those in bench sciences, noted delays in research for long periods over several months due to limited access to laboratories and offices. Inability to conduct experiments was a major factor reducing research productivity, which also affected generating teaching materials based on research results. Participants encountered canceled or halted field trips for data collection due to COVID-19 regulations including lockdown and ban of domestic/international travel. Recruitment of new staff in laboratories and human participants for research was limited to reduce risk of disease spread during the pandemic. Many participants expressed that delayed recruitment interrupted original research plans. For example, a respondent working with human participants noted that the complete shutdown of their research led to establishing an entirely new project and slowed their productivity (ID274, USA). Participants expressed suffering from canceled or postponed international research training. The reduction in the number of researchers in research facilities led to perceptions that workload increased for the remaining personnel who had access to laboratories and had to perform the research.

##### Example response

“As an experimental scientist and P.I., the limited accessibility of lab resources and recruitment of talented personnel reduced the scientific productivity significantly (ID266, USA).”

#### Shortage of research equipment

Among participants engaged in research, significant delays in research products (e.g., plasticware, personal protective equipment, reagents) slowed research, especially for those involving bench experiments.

##### Example response

“We have had to halt some experiments because we do not have necessary materials and I am a year behind intended publication schedule (ID1115, Australia).”

#### Canceled grants and delayed publication

Grants and funding for research were canceled or decreased during the pandemic leading to significantly reduced research outputs. Some participants perceived decreased success rate for new funding during the pandemic. Some also reported slower review processes for scientific journals affecting their publications. Participants expressed that finding reviewers for some journals took longer and the review process was extended. Some noted that some journals received a significantly increased number of articles and their papers that could have been published in the past have been rejected and the level of the journals they can publish in has lowered.

##### Example response

“The most noticeable effect was the time required to find reviewers in some journals, the review process was extended (ID1041, Finland).”

### Theme 2: Changes in responsibilities

#### Teaching burdens and online supervision

Time for teaching increased for many participants during the pandemic as teaching transitioned to online. Restructuring online courses and adapting to online systems required significant time for many participants. A few participants mentioned that no or little support and guidance were provided by their institutions despite the increased responsibility for organizing new course timetables and online teaching. Some participants were given additional (online) courses to teach. Some described changes in scheduling and distorted timelines due to increased teaching responsibilities as “abrupt” (participant number ID383, Poland) and “brutal” (ID1169, USA). Many expressed difficulties in teaching and supervising.

Online teaching was less effective and more time-consuming for many participants, and teaching quality was negatively affected. A participant expressed that remote training was more difficult while the needs for training and emotional support for students and staff increased during the pandemic (ID1635, USA). Other major challenges for teaching and supervision included time differences for students in different countries, unstable internet connection, lack of direct interactions, and distractions. On the contrary, some participants found that transitioning to online teaching increased productivity. One participant mentioned that the increased teaching efforts to generate new educational materials for remote teaching in the first year of the pandemic improved teaching in 2021 (ID 2209, South Korea).

##### Example response

“Professors had to organize new course timetables and online teaching with little or no help from administrative personnel and very poor guidelines (ID1235, Spain).”

#### No or little time for research

As duties and time for teaching, mentoring, or administrative work increased, time for research decreased for many participants. Some expressed that their university still expected all academic staff to have publications while access to laboratories was restricted.

##### Example response

“Grant applications were delayed due to lack of time but also due to reduced contact with colleagues as we are all working at home (ID354, USA).”

#### Increased burden for child/eldercare and domestic work

Childcare and housework became intertwined with work during the pandemic. This was described as a “juggle” between work and caregiving by some participants. A participant described the effect on productivity as devastating (ID1291, Italy). Another reported that having to work from home and supervise school-age children hindered her from working efficiently (ID934, Greece). The identified reasons for increased childcare included difficulties in hiring childcare workers, closure of daycare facilities, and homeschooling due to school closures. Parents noted markedly increased stress from balancing work and childcare simultaneously at home. While some participants mentioned difficulties of juggling work and childcare, others described aspects of productivity that were affected by changed caregiving responsibilities. For example, one mentioned that her publication outputs were lower than normal (ID1260, Australia) due to leading children’s homeschooling and working from home in conjunction with increased household duties. Another mentioned that working from home with kids at home reduced his productivity (ID1167, USA).

We found that the number of work hours increased for some participants with children at home, but decreased for others. Some participants worked at night to compensate for lost productivity caused by the increased time for childcare, which deteriorated psychological and physical health. Others had decreased work hours due to struggles with mental and emotional capacity to focus on work and stress from caregiving.

Relatively more female parents than male parents mentioned increased childcare burdens (Table 2), but we did not find significant differences in the content or degrees of expression of hardships for childcare between these groups. A few participants wrote that they had equal distributions of childcare with their partner/spouse during the pandemic.

**Table 2:** The number and percentage of participants mentioning each key text regarding caregiving and domestic work in survey responses, for parents of children <18 years living at home.

Text	Total participants who were mothers (*N* = 442)		Total participants who were fathers (*N* = 614)	
	*N*	Percent (%)	*N*	Percent (%)
Child	31	7.01	16	2.61
Childcare	34	7.69	25	4.07
Children	41	9.28	26	4.23
Son	3	0.68	1	0.16
Daughter	1	0.23	0	0.00
Baby	1	0.23	1	0.16
Kid	31	7.01	14	2.28
Elder	2	0.45	0	0.00
Daycare	15	3.39	8	1.30
School^a^	43	9.73	25	4.07
Family	23	5.20	14	2.28
Parent^a^	3	0.68	3	0.49
Mother	2	0.45	1	0.16
Father	0	0.00	2	0.33
Dad	0	0.00	0	0.00
Mom	2	0.45	9	0.00
Household	6	1.36	1	0.16
Chore	0	0.00	3	0.49
Domestic^b^	3	0.68	2	0.33

^a^Stemmed words for each text were included in the search (e.g., “school”, “schooling”; “parent”, “parenting”).

^b^Only responses referring to “domestic work” were counted.

Increases in domestic work and caregiving for family other than children were identified as factors that made work difficult during the pandemic. There were a few single participants that reported a higher workload due to performing the work of their co-workers with children and families.

##### Example response

“The most demanding was the online learning of children, me (and my wife and both parents) had to partly substitute the teachers (ID1561, Czech Republic).”

#### Increased number of online meetings

The significant increase in the number and time of online meetings reduced the time that could be spent on other tasks. Some participants described the increased burden of online meetings as depressing, exhausting, and stressful. A few participants also mentioned that the duration of online meetings was very long (e.g., in one instance, over 6 h) and sometimes took a place at night. On the contrary, a few participants favored the increase in online meetings as it kept meeting times concise and efficient and increased work efficiency and opportunities for collaboration.

##### Example response

*“*Several meetings have more online making it easier to collaborate with cross-border teams (ID2381, Nigeria).”

### Theme 3: Deteriorated health

#### Deterioration of physical health

Working from home, which was isolating, and working long hours at home were associated with various health issues including office syndrome, lack of sleep and breaks, and fatigue for many participants. Some participants also experienced disruption of work after COVID-19 infection. A few participants noted that working in masks made them feel fatigued quicker. Some participants who mentioned physical health issues also expressed that their productivity was affected as they were exhausted or fatigued.

##### Example response

“Decreased overall work productivity, I lost days when I was sick. I couldn’t keep up with emails, notes, etc. I’m still not fully caught up months later (ID773, USA).”

#### Deterioration of mental health

Mental health issues at work and home during the pandemic were found to be harmful to scientific productivity for many participants. Many experienced burnout from working overtime despite their increased productivity. Slowed research, fewer interactions, high work demand, altered priorities of tasks, and minimal administrative support affected motivation. The lockdown and confinement at home were associated with a lack of motivation and decreased productivity for some participants. A participant expressed that stress and depression decreased her scientific creativity and focusing power (ID2534, Romania). Another mentioned that a decline in enthusiasm and work mood slightly reduced his productivity (ID2507, Germany).

While many participants said that they were affected mentally (e.g., depression, anxiety, stress, worries) due to work-related challenges during the pandemic, some expressed that anxiety and fear directly related to COVID-19 interfered with work productivity. Uncertainty about COVID-19 and job security also caused stress and anxiety among some participants.

##### Example response

“Overall, the lack of job security and increased workload has led to increased anxiety and loss of motivation (ID18, Bangladesh).”

### Theme 4: Insufficient at-home work environment

#### Blurred boundary between work and life

Many participants working from home noted difficulties in dividing time between work and life and often worked overtime. Many participants reported working more hours by working from home and expressed their fatigue and burnout from working overtime (see Theme 3). The increased time for work did not always increase productivity due to distractions affecting concentration, more administrative processes, or interruptions of work routines by priority tasks due to the pandemic. Some participants noted that this situation made it hard to concentrate on work and affected focus and performance.

While some participants expressed fatigue and frustration caused by the high demand for “being always available at home for work”, a few viewed this situation as positive leading to increased productivity because colleagues and collaborators are more available with remote working. For example, one wrote, “Increased productivity as colleagues and collaborators are more available online (ID526, Switzerland).”

##### Example response

“Since I work at home, my superiors expect me to be always available, and I end up having trouble keeping regular working hours. I feel tired often (ID1159, Bulgaria).”

#### Insufficient workspace and/or set-up

Some participants encountered challenging situations including unsatisfactory home workspace or unstable internet connection. Not having office supplies at home was also mentioned as a reason for inefficient homework environment by a few participants.

##### Example response

“Working from home without having a proper workspace resulted in inefficiency (ID2448, Canada).”

#### Lack of in-person interactions

Insufficient communication due to remote working during the lockdown was found as a sub-theme negatively affecting scientific productivity. Participants responded that communicating remotely was less efficient and more time-consuming than face-to-face interactions. In terms of staffing and support, some participants expressed that communication slowed as most staff were working from home and conversation over emails took longer than in-person interactions. Some participants noted that opportunities for open and creative discussion for work and sharing research outputs decreased due to remote working and a lack of formal events such as conferences and seminars. They mentioned that planned research projects were delayed due to decreased in-person collaborations. Some participants mentioned difficulties in supervising remotely. Physical disconnection from colleagues and having less or no face-to-face interactions affected productivity with feelings of isolation and reduced motivation for some participants. Some early-career scientists expressed hardships in socializing and getting to know new colleagues through remote working environments, which slowed their adaptation to new work and positions.

##### Example response

“E-mail-based supervision is time-consuming (ID922, Estonia).”

### Theme 5: Increased flexibility and other positive aspects

#### Teaching environment

Participants also identified positive impacts on work during the pandemic. A few participants favored transitioning to online teaching. Some stated that remote teaching was more efficient than in-person classes for some activities involving computer science. Also, a participant mentioned that utilizing online teaching techniques and not having to book classrooms added flexibility.

##### Example response

“I have more flexibility in teaching and teaching methods as I don’t need to book lecture halls and can experiment with different techniques (ID561, Sweden).”

#### Grants for COVID-19 research

A few respondents in fields directly relevant to COVID-19 topics reported that they have been “more productive in terms of publications and grants (ID1721, USA)” with “Fantastic increase in productivity - covid research (ID1471, USA).” Some mentioned that having more funds and grants for COVID-19 research led to valuable research experience and publications.

##### Example response

“Actually (the pandemic) increased scholarly activities as they expanded to include SARS-CoV-2 and COVID work. They are directly related to our clinical and public health research interests (ID1157, Canada).”

#### Alternative methods

While the previous sub-theme was about new research opportunities related to COVID-19, this sub-theme is about efforts to change the way research is conducted. Many participants suffering from limited access to laboratories reported switching to different study methodologies or activities. The time used for conducting laboratory experiments was instead spent writing scientific papers and grant applications. Applying study designs such as online surveys that do not require face-to-face contact, re-analyzing old experimental data, and writing review articles were reported as alternative research activities during the pandemic to maintain productivity. These altered approaches resulted in increased number of publications for some participants but not for all.

Some participants who had applied alternative research methods and maintained publication productivity were worried about their future research productivity, such as the inability to collect new data.

##### Example response

“Due to the lack of experimental studies my work has primarily focused on surveys and other types of research that does not require direct contact with participants (ID1562, Netherlands).”

#### Fluidity of workday

This sub-theme summarizes the preference of many participants for the flexibility of working from home without commuting or traveling. Increases in the number of work hours were described as positive changes for work during the pandemic for these participants. The flexibility of working from home was described as “excellent (ID1011, Australia)” and “a positive game changer (ID1338, Germany)” for saving time and increasing productivity. Many participants reported that their time increased for writing and publishing papers, focusing, and conducting research due to flexible schedules. Some participants described the working environment at home as “distraction-free (ID387, Germany)” and “quieter (ID1626, USA).”

Some participants mentioned that the reduction of unnecessary meetings or chats provided additional time for research and other work. Furthermore, a few participants commented that they could select when to interact with staff or students via emails or interact with collaborators in different time zones via online meetings while working from home. A few participants responded that they saved time due to the prohibition of after-work dinner parties. This positive voice for eliminating “unnecessary” gathering after work, an extension of work, contrasted with colleagues who experienced isolation (see Theme 3).

##### Example response

“The COVID-19 pandemic has increased work productivity. It has saved time from transportation; provided flexibility for interaction through internet platforms at my convenience; enabled watching a number of webinars and interact with various communities that would not be possible through face-to-face interaction (ID87, Greece).”

### Results of word cloud analysis

Results of work cloud analysis are shown in Fig. 1. The most frequently mentioned word was “work” followed by “time”, “research”, “productivity”, “home”, “increased”, and “pandemic”. The participants often mentioned “working”, “teaching”, “online”, and “students”, which are relevant for teaching, mentoring, and supervision. Our participants also frequently mentioned “lab”, “meetings”, “colleagues”, “access”, “restrictions”, “travel”, and “remote”. Terms relevant for caregiving responsibility including “care”, “childcare”, “child”, “children”, “kids”, and “family” were also identified and so were terms for health and life such as “mental”, “stress”, “health”, and “life”.

### Results of text search analysis

Word search analysis examined how female and male participants expressed their experiences regarding caregiving for children, family, and domestic work during the pandemic. Results found that the burden from increased duties for childcare and domestic work during the COVID-19 pandemic and their negative effects on scientific productivity may be higher in mothers than fathers given that mothers mentioned select keywords related to children and domestic work more frequently than fathers (Table 2).

## Discussion

Although research is needed on the impacts of the COVID-19 pandemic on scientific productivity (Biswakarma et al., 2021), studies remained limited. Studies on the impacts of COVID-19 on scientific productivity often did not examine gender disparities and inequitable changes in caregiving by gender and status as a parent (Staniscuaski et al., 2021). A number of studies quantitatively compared scientific publications and authorship by gender, with inconsistent findings (See Supplementary Table S5). Some studies compared the changes in the number of work hours between men and women scientists. These studies did not examine the contributing factors from the work routines and environment on gender differences. Some studies focused on the experiences of scientists during the pandemic, with a focus on only one type of scientific activity such as teaching and research. Evidence of the affected work environment for administrative activities among scientists during the pandemic is scarce.

Given these characteristics of previous studies, this current study contributes to identifying in-depth challenges in various work activities among scientists during the COVID-19 pandemic, including gender differences, using information collected from over 132 countries using a six-language survey with an open-ended question. Moreover, by comprehensively summarizing and categorizing the impacts of COVID-19 on scientific labors in addition to changes in work and daily life, this research contributed to understanding of the inequality in the burden of household labor during the pandemic, which is gender inequality in scientific performance during the pandemic.

Our findings indicate conflicting impacts across participants. Some scientists may have increased time for research due to decreased teaching burden, whereas others may have had less time for research due to increased burdens for teaching, administration, or grant applications. Many participants experienced conflicting demands for teaching, research, and administration due to COVID-19. The varying situations and challenges in scientific fields (e.g., some participants expressing frustration with remote work while others found it helpful) indicate that support and policies for scientists during times of crisis should be flexibly applied and recognize the different impacts rather than being applied strictly and uniformly.

A previous study discussed that many faculty were unfamiliar with online teaching software prior to the pandemic and that many universities were unprepared to provide proper guidance and training for transitioning to remote learning (Coyne et al., 2020). Similarly, many of our participants noted that time for teaching increased during the pandemic as teaching transitioned to online. Faculty experienced a remarkable loss of in-person interactions with students and colleagues while teaching remotely (Colclasure et al., 2021). For some scientists involved in research and teaching, the time to pursue research was modified due to the increased demands for teaching and service during the pandemic (Malisch et al., 2020). However, a few participants in our study found online teaching to be more efficient to reach students and perform computer assignments. It is likely that demand for teaching would take priority when educational facilities are restricted and in-person learning becomes infeasible in future crises. Thus, educational institutions should establish rapid action guidelines for teaching transitions. Another challenge during pandemics is altered quality of teaching, which can affect faculty’s promotion. Institutions should find appropriate measures of teaching quality and effectiveness that account for these unique challenges (Sotto-Santiago et al., 2021).

While our focus was to find a broad spectrum of the COVID-19 impacts on scientific work productivity, the impacts of COVID-19 and institutional solutions constantly change overtime based on the severity of the disease transmission trend. Thus, we note that the themes found in our research and the obstacles from them perceived among scientists can considerably vary by the time of pandemic and the level of institutional reactions to the pandemic. This indicates the need of continuous efforts to recognize scientists’ difficulties during and after the pandemic by the institutions and leaders.

We found that time for research, teaching, mentoring, and administrative work was diverted to other obligations at home during the pandemic, more so for some participants than others. Participants with pre-school or school-age children noted struggles to find time and space for work while performing increased caregiving for their children during the pandemic. Especially, mothers expressed such struggles more than fathers. Many previous studies showed that school closures and stay-at-home orders amplified gender differences in productivity by disrupting both work and life for many researchers (Andersen et al., 2020; Malisch et al., 2020; Breuning et al., 2021). The blurred boundaries between work and responsibilities at home led to significant mental health issues such as stress and burnout among many participants. Institutional supports are critically needed, especially to diminish gender differences of academic productivity caused by unequitable division of responsibilities at work and home. Some policies for reducing gender inequity include creating an infrastructure for identifying and providing childcare resources, accommodating flexible working arrangements, increasing funding for opportunities for certain groups, monitoring sex breakdown in promotion and tenure, and monitoring teaching load and service (Cardel et al., 2020; Minello et al., 2021).

Our results found that many scientists suffered from health issues including lack of sleep and exercise, burnout, fatigue, and deterioration of mental health. Increased work burdens, uncertainty regarding the pandemic, and lack of interactions with others were associated with mental health issues for many participants, as also shown in previous studies (Camerlink et al., 2021; Colclasure et al., 2021). Some participants were worried and fearful about long-term effects of the pandemic on their productivity and career progress, similarly observed in previous studies (Minello et al., 2021). A previous study using surveys and interviews found that a lack of face-to-face interactions with students increased personal stress and anxiety among faculty at institutions primarily for undergraduate students (Colclasure et al., 2021). These results suggest that university and research institutions should find formats to maintain connections during lockdowns with technical infrastructure and supports for mental health (Sahu, 2020).

Some positive aspects of the changed work environment during the pandemic were favored by some scientists. A recent study in the UK presented several benefits in the early stages of the pandemic including working from home thereby avoiding commuting time and increasing productivity and work-life balance (Jackman et al., 2021). Similarly, some of our participants mentioned that time saved from not commuting can be used for mental and physical health. These findings may help identify key intervention points; accommodating flexible work arrangements (Cardel et al., 2020) and providing technical support for effective supervision and communication for remote working may positively impact scientists’ productivity during a pandemic.

Scientists may engage in more creative scientific works when away from laboratories during a pandemic by spending time to read the latest literature, carefully plan experiments, and write grant proposals and manuscripts (Buchanan et al., 2021). However, over longer periods of restrictions, some scientists would suffer from a lack of new data and access to key resources. Our results imply that even scientists who managed to effectively publish and conduct research during the pandemic were worried about long-term effects on their productivity.

This study has several limitations. As we targeted scientists in STEMM fields, our findings may not be generalizable for other disciplines. As the survey introduction and consent form stated our goal to investigate gender differences in scientific productivity and mental health during the pandemic, those who were interested in these topics may have been more likely to participate, affecting the findings. Also, our survey participants are more likely to have research-related positions than government scientists or instructors due to our recruitment process using scientific articles from publication databases. Second, due to the limitation of a thematic analysis, we were not able to compare the findings of this research among subgroups of geographical region, position, employment status (e.g., full-time, part-time), or tenure status, which may be a factor of different impacts of the COVID-19 pandemic on productivity.

The thematic analysis of this research is an effective and established method (Braun and Clarke, 2006; Kiger and Varpio, 2020) to provide a summary of the themes among the recruited study participants. Additional results on other aspects of the survey include a quantitative analysis, which we have previously provided elsewhere (Heo et al., 2022). Here, we focus on a qualitative, thematic analysis, and also report a summary table of the percentage of our study participants reporting each of the found themes among different geographical regions in supplementary materials (Supplementary Table S4). This type of analysis can indicate the need for future studies with quantitative analyses for further understanding of disparities of the COVID-19 impacts on productivity by regions, but our results should be interpreted cautiously. In addition, qualitative analyses can be criticized as they can be more subjective than quantitative studies and there is a degree of subjectivity in all states of the analysis, which may lead to opportunities for bias (Chapman et al., 2015; Roberts et al., 2019). To minimize such potential bias, two investigators independently drafted potential codes and then performed inter-rater comparisons and discussions iteratively reviewing and moving them until an agreement between the two investigators as to what determined sufficient demonstration of a true theme became evident. Despite our efforts for the reproducibility of analyses, we note that it is important to recognize the nature of the thematic analysis with regard to the characteristics of the respondent group and organizational and geographical context (Chapman et al., 2015).

Moreover, we recognize that the distinction between sex and gender is critically important and that there are multiple genders (Torgrimson and Minson, 2005; Reisner et al., 2016; Peters and Norton, 2018; Spizzirri et al., 2021). However, the words “sex” and “gender” are commonly used interchangeably in countries where there are currently no separate words for sex and gender. Thus, we were challenged to address these concepts across six languages and the cultures of 132 countries where these concepts vary considerably compared to Western usage (Riley, 1997; Abbey et al., 2004; Clayton and Tannenbaum, 2016; Schiebinger and Stefanick, 2016; Peters and Norton, 2018; Morgenroth et al., 2021). The term “sex” used in this paper likely represented sex for some participants and gender for others, but the impacts of COVID-19 on the academic fields may differ between sex and genders. Therefore, substantial future studies are required with more detailed subgroups for sex and gender, with language specific to the culture.

Although we used a published method and received IRB approval, our survey recruitment process raised concerns for a few email recipients regarding privacy issues as they had not initiated contact with us before receiving an unsolicited email. Thus, we recommend different survey recruitment processes in future studies. Our study design was cross-sectional, which is limited for validating temporal relationships. The word cloud analysis included some words and phrases translated from other languages, which may have affected findings. Although we provided a definition of academic activities including research, teaching, mentoring, and administrative service in our questionnaire, the definition of productivity could vary among participants. Therefore, the answers in our survey were based on academic productivity self-defined by our participants and their perceptions of their productivity.

This study has several strengths. Our survey reached various populations using six languages. As a result, we had a variety of study participants in terms of position, rank, career stage, tenure status, major, and characteristics of work (e.g., “wet” and “dry” science), with participants from 132 countries. Previous results are limited to the quantitative analysis using publications as a measure for productivity changes during the pandemic, which is likely insufficient to reflect the productivity of other scientific work other than publishing articles. These previous studies often lacked consideration of disparities by gender and parental status of the impact of the pandemic on performance (Staniscuaski et al., 2021). Furthermore, in most previous studies, results for changes in scientific performance were based on responses from the US- and European institutions and were scarce for other regions (Webber, 2012). A contribution of our research is that it summarized the intersecting vulnerabilities by gender and status as a parent in scientific fields and better insight into the systematic changes of scientific institutions during a global crisis. Second, we investigated the perceived effect of the pandemic on scientific productivity and self-reported mental health status for faculty in STEMM fields; the body of scientific literature for pandemic-related obstacles and mental health effects was relatively smaller for faculty compared to students (Breuning et al., 2021). Through an open-ended question and systematic thematic analysis, we allowed participants’ to share their experiences of how the pandemic affected their scientific endeavors.

In summary, this study provides important insights into the struggles and opportunities scientists can face during a global health crisis, showing that the impacts varied widely, even for the same change in work (e.g., remote working increased productivity for some and lowered productivity for others). The adverse impacts of the COVID-19 pandemic on academic productivity are likely to last long after the pandemic. Our findings suggest that academic communities need to recognize the different impacts of the pandemic on scientists and ensure flexibility in policies for promotion and job security. Leaders of universities and research institutions should explore the obstacles for scientists and review their crisis management plans to best support scientists for productivity and career, recognizing that scientists will experience these challenges differently. Institutional efforts are imperative to avoid widening gender gaps in academia during and after the pandemic.

## Supplementary information

Supplementary Information

## Data Availability

The data that support the findings of this study are available on request from the corresponding author. The data are not publicly available as the data contain potentially sensitive information that could compromise research participant privacy and the study participants did not consent to share any data publicly.
