# Supplementary material for: Impacts of the COVID-19 pandemic on scientists’ productivity in science, technology, engineering, mathematics (STEM), and medicine fields
Source: Humanit Soc Sci Commun. 2022 Dec 3;9(1):434. doi: 10.1057/s41599-022-01466-0 (PMC9734604; doi:10.1057/s41599-022-01466-0)
Supplement: Supplementary file 1 — Supplementary Information [file 41599_2022_1466_MOESM1_ESM.docx]

Supplemental materials

Impacts of the COVID-19 pandemic on scientists’ productivity in science, technology, engineering, mathematics (STEM), and medicine fields

Seulkee Heo^1,*^, Alisha Yee Chan^2, 1^, Pedro Diaz Peralta^3, 1^, Lan Jin^4^, Claudia Ribeiro Pereira Nunes^5, 1^, Michelle L. Bell^1^

^1^ School of the Environment, Yale University, New Haven, CT, USA

^2^ Department of Chemical and Environmental Engineering, School of Engineering and Applied Science, Yale University, New Haven, CT, USA

^3^ Administrative Law Department, School of Law, Universidad Complutense de Madrid, Madrid, Spain

^4^ School of Public Health, Yale University, New Haven, CT, USA

^5^ Graduate Program in Law, School of Law, Federal University of Amazon, Manaus, Amazonas, Brazil

^*^Author to whom correspondence should be addressed ([seulkee.heo@yale.edu](mailto:seulkee.heo@yale.edu)).

<Contents>

Table S1. Standards for Reporting Qualitative Research (SRQR)^a^.

Table S2. Characteristics of participants.

Table S3. Supporting quotes for each theme and sub-theme found in the survey data.

Table S4. The number of participants who reported each of the found themes in this research by geographical regions (n=2548).

Figure S1. Initial thematic map, showing 8 potential main themes - Version 1.

Figure S2. Initial thematic map, showing 8 potential main themes - Version 2.

Table S1. Standards for Reporting Qualitative Research (SRQR)^a^.

| No. | Topic | Item | Page |
| --- | --- | --- | --- |
| Title and abstract | | | |
| S1 | Title | Concise description of the nature and topic of the study Identifying  the study as qualitative or indicating the approach (e.g., ethnography,  grounded theory) or data collection methods (e.g., interview, focus  group) is recommended | Page 1 |
| S2 | Abstract | Summary of key elements of the study using the abstract format of  the intended publication; typically includes background, purpose,  methods, results, and conclusions | Page 1–2 |
| Introduction | | | |
| S3 | Problem formulation | Description and significance of the problem/phenomenon studied; review of relevant theory and empirical work; problem statement | Page 4–5 |
| S4 | Purpose or research question | Purpose of the study and specific objectives or questions | Page 5 |
| Methods | | | |
| S5 | Qualitative approach and research paradigm | Qualitative approach (e.g., ethnography, grounded theory, case study, phenomenology, narrative research) and guiding theory if appropriate; identifying the research paradigm (e.g., postpositivist, constructivist/  interpretivist) is also recommended; rationale^b^ | Page 6 (Thematic analysis for the survey data) |
| S6 | Researcher characteristics and reflexivity | Researchers’ characteristics that may influence the research, including personal attributes, qualifications/experience, relationship with  participants, assumptions, and/or presuppositions; potential or actual interaction between researchers’ characteristics and the research questions, approach, methods, results, and/or transferability | Page 3 (Survey recruitment) |
| S7 | Context | Setting/site and salient contextual factors; rationale^b^ | Page 5 (Survey recruitment) |
| S8 | Sampling strategy | How and why research participants, documents, or events were selected; criteria for deciding when no further sampling was necessary (e.g., sampling saturation); rationale^b^ | Page 5 (Survey recruitment) |
| S9 | Ethical issues pertaining to human subjects | Documentation of approval by an appropriate ethics review board and participant consent, or explanation for lack thereof; other confidentiality and data security issues | Page 5–6 (Survey recruitment) |
| S10 | Data collection methods | Types of data collected; details of data collection procedures including (as appropriate) start and stop dates of data collection and analysis, iterative process, triangulation of sources/methods, and modification of procedures in response to evolving study findings; rationale^b^ | Page 5–6 |
| S11 | Data collection instruments and technologies | Description of instruments (e.g., interview guides, questionnaires) and devices (e.g., audio recorders) used for data collection; if/how the instrument(s) changed over the course of the study | Page 5 (Survey recruitment) |
| S12 | Units of study | Number and relevant characteristics of participants, documents, or events included in the study; level of participation (could be reported in results) | Supplemental Table S1 |
| S13 | Data processing | Methods for processing data prior to and during analysis, including transcription, data entry, data management and security, verification of data integrity, data coding, and anonymization/deidentification of  excerpts | Page 6 (Thematic analysis for the survey data) |
| S14 | Data analysis | Process by which inferences, themes, etc., were identified and developed, including the researchers involved in data analysis; usually references a specific paradigm or approach; rationale ^b^ | Page 6–8 (Thematic analysis for the survey data, word cloud analysis, text search analysis) |
| S15 | Techniques to enhance trustworthiness | Techniques to enhance trustworthiness and credibility of data analysis (e.g., member checking, audit trail, triangulation); rationale^b^ | Page 6 (2nd paragraph of Thematic analysis for the survey data) |
| Results/findings | | | |
| S16 | Synthesis and interpretation | Main findings (e.g., interpretations, inferences, and themes); might include development of a theory or model, or integration with prior research or theory | Page 8–20 |
| S17 | Links to empirical data | Evidence (e.g., quotes, field notes, text excerpts, photographs) to substantiate analytic findings | Page 8–20 |
| Discussion | | | |
| S18 | Integration with prior work, implications,  transferability, and contribution(s) to the field | Short summary of main findings; explanation of how findings  and conclusions connect to, support, elaborate on, or challenge  conclusions of earlier scholarship; discussion of scope of application/  generalizability; identification of unique contribution(s) to scholarship  in a discipline or field | Page 21–23 (Discussion) |
| S19 | Limitations | Trustworthiness and limitations of findings | Page 23 |
| Other | | | |
| S20 | Conflicts of interest | Potential sources of influence or perceived influence on study conduct  and conclusions; how these were managed | Separate title page (page 2) |
| S21 | Funding | Sources of funding and other support; role of funders in data  collection, interpretation, and reporting | Separate title page (page 2) |

^a^ The authors created the SRQR by searching the literature to identify guidelines, reporting standards, and critical appraisal criteria for qualitative research; reviewing the reference lists of retrieved sources; and contacting experts to gain feedback. The SRQR aims to improve the transparency of all aspects of qualitative research by providing clear standards for reporting qualitative research.

^b^ The rationale should briefly discuss the justification for choosing that theory, approach, method, or technique rather than other options available, the assumptions and limitations implicit in those choices, and how those choices influence study conclusions and transferability. As appropriate, the rationale for several items might be discussed together.

Table S2. Characteristics of participants.

|  | Total  (*n* = 2548) | English  (*n* = 2184) | Spanish  (*n* = 93) | Portuguese  (*n* =58) | Korean  (*n* = 77) | Chinese  (n = 123) | Japanese  (*n* = 13) |
| --- | --- | --- | --- | --- | --- | --- | --- |
| Sex/gender^a^ |  |  |  |  |  |  |  |
| Male | 1421 (55.8) | 1170 (53.6) | 60 (64.5) | 26 (44.8) | 61 (79.2) | 93 (75.6) | 11 (84.6) |
| Female | 1103 (43.3) | 990 (45.3) | 32 (34.4) | 32 (55.2) | 16 (20.8) | 30 (24.4) | 2 (15.4) |
| Other | 25 (0.9) | 24 (1.1) | 1 (1.1) | 0 (0.0) | 0 (0.0) | 0 (0.0) | 0 (0.0) |
| Position |  |  |  |  |  |  |  |
| Postdoc | 630 (24.7) | 562 (25.7) | 1 (1.1) | 6 (10.3) | 8 (10.4) | 5 (4.1) | 0 (0.0) |
| Researcher | 581 (22.8) | 477 (21.9) | 43 (46.7) | 11 (19.0) | 27 (35.1) | 21 (17.1) | 2 (15.4) |
| Instructor | 94 (3.7) | 79 (3.6) | 1 (1.1) | 3 (5.2) | 1 (1.3) | 8 (6.5) | 2 (15.4) |
| Assistant professor | 330 (13.0) | 287 (13.2) | 16 (17.4) | 11 (19.0) | 10 (13.0) | 5 (4.1) | 1 (7.7) |
| Associate professor | 465 (18.3) | 381 (17.5) | 18 (19.6) | 12 (20.7) | 9 (11.7) | 44 (35.8) | 1 (7.7) |
| Professor | 630 (24.7) | 562 (25.7) | 7 (7.6) | 8 (13.8) | 17 (22.1) | 31 (25.2) | 5 (38.5) |
| Dean | 82 (3.2) | 69 (3.2) | 3 (3.3) | 2 (3.5) | 1 (1.3) | 7 (5.7) | 0 (0.0) |
| Other | 155 (6.1) | 139 (6.4) | 3 (3.3) | 5 (8.6) | 4 (5.2) | 2 (1.6) | 2 (15.4) |
| Status as a parent (children <18 years) |  |  |  |  |  |  |  |
| Yes | 1057 (41.5) | 878 (40.2) | 29 (31.2) | 18 (31.0) | 40 (52.0) | 84 (68.3) | 8 (61.5) |
| No | 1491 (58.5) | 1306 (59.8) | 64 (68.8) | 40 (69.0) | 37 (48.1) | 39 (31.7) | 5 (38.5) |
| Region^b^ |  |  |  |  |  |  |  |
| Africa | 93 (3.7) | 93 (4.3) | 0 (0.0) | 0 (0.0) | 0 (0.0) | 0 (0.0) | 0 (0.0) |
| Asia | 426 (16.9) | 233 (10.8) | 0 (0.0) | 0 (0.0) | 65 (84.4) | 115 (94.3) | 13 (100.0) |
| Caribbean | 5 (0.2) | 5 (0.2) | 0 (0.0) | 0 (0.0) | 0 (0.0) | 0 (0.0) | 0 (0.0) |
| Europe | 863 (34.2) | 817 (37.8) | 33 (35.9) | 9 (15.5) | 3 (3.9) | 1 (0.8) | 0 (0.0) |
| North America | 845 (33.5) | 831 (38.4) | 2 (2.2) | 0 (0.0) | 9 (12.7) | 3 (2.5) | 0 (0.0) |
| Oceania | 132 (5.2) | 132 (6.1) | 0 (0.0) | 0 (0.0) | 0 (0.0) | 0 (0.0) | 0 (0.0) |
| South America | 156 (6.2) | 50 (2.3) | 57 (62.0) | 49 (84.5) | 0 (0.0) | 0 (0.0) | 0 (0.0) |
| Other | 6 (0.2) | 3 (0.1) | 0 (0.0) | 0 (0.0) | 0 (0.0) | 3 (2.5) | 0 (0.0) |
| Field |  |  |  |  |  |  |  |
| Agriculture/natural resources | 192 (7.5) | 171 (7.8) | 11 (12.0) | 1 (1.7) | 3 (3.9) | 4 (3.3) | 2 (15.4) |
| Astronomy/astrophysics | 35 (1.4) | 30 (1.4) | 1 (1.1) | 0 (0.0) | 1 (1.3) | 3 (2.4) | 0 (0.0) |
| Biology | 579 (22.7) | 500 (22.9) | 19 (20.7) | 20 (34.5) | 12 (15.6) | 24 (19.5) | 4 (30.8) |
| Chemistry | 207 (8.1) | 179 (8.2) | 8 (8,7) | 3 (5.2) | 5 (6.5) | 12 (9.8) | 0 (0.0) |
| Computer science | 166 (6.5) | 140 (6.4) | 6 (6.5) | 2 (3.4) | 11 (14.3) | 6 (4.9) | 1 (7.7) |
| Engineering | 339 (13.3) | 287 (13.1) | 12 (13.0) | 4 (6.9) | 17 (22.1) | 16 (13.0) | 3 (23.1) |
| Environment/earth science | 273 (10.7) | 242 (11.1) | 6 (6.5) | 4 (6.9) | 3 (3.9) | 13 (10.6) | 4 (30.8) |
| Geology | 47 (1.8) | 39 (1.8) | 3 (3.3) | 0 (0.0) | 2 (2.6) | 2 (1.6) | 1 (7.7) |
| Interdisciplinary research | 251 (9.9) | 216 (9.9) | 9 (9.8) | 5 (8.6) | 9 (11.7) | 11 (8.9) | 0 (0.0) |
| Mathematics | 130 (5.1) | 109 (5.0) | 6 (6.5) | 2 (3.4) | 6 (7.8) | 7 (5.7) | 0 (0.0) |
| Medicine/public health | 993 (39.0) | 866 (39.7) | 29 (31.5) | 24 (41.4) | 25 (32.5) | 48 (39.0) | 1 (7.7) |
| Physics | 154 (6.0) | 131 (6.0) | 5 (5.4) | 6 (10.3) | 4 (5.2) | 6 (4.9) | 2 (15.4) |
| Zoology / animal science | 76 (3.0) | 66 (3.0) | 4 (4.3) | 2 (3.4) | 0 (0.0) | 3 (2.4) | 1 (7.7) |
| Other | 261 (10.2) | 218 (10.0) | 12 (13.0) | 3 (5.2) | 0 (0.0) | 13 (10.6) | 2 (15.4) |
| Age (years) |  |  |  |  |  |  |  |
| 20 – 24 | 1 (0.0) | 1 (0) | 0 (0.0) | 0 (0.0) | 0 (0.0) | 0 (0.0) | 0 (0.0) |
| 25 – 29 | 68 (2.7) | 64 (2.9) | 1 (1.1) | 2 (3.4) | 0 (0.0) | 1 (0.8) | 0 (0.0) |
| 30 – 34 | 228 (8.9) | 190 (8.7) | 4 (4.3) | 7 (12.1) | 5 (6.5) | 22 (17.9) | 0 (0.0) |
| 35 – 39 | 382 (15.0) | 326 (14.9) | 8 (8.6) | 8 (13.8) | 13 (16.9) | 27 (22.0) | 0 (0.0) |
| 40 – 44 | 422 (16.6) | 343 (15.7) | 17 (18.3) | 6 (10.3) | 18 (23.4) | 25 (20.3) | 13 (100.0) |
| 45 – 49 | 349 (13.7) | 294 (13.5) | 18 (19.4) | 9 (15.5) | 12 (15.6) | 16 (13.0) | 0 (0.0) |
| 50 – 54 | 334 (13.1) | 275 (12.6) | 23 (24.7) | 8 (13.8) | 12 (15.6) | 16 (13.0) | 0 (0.0) |
| 55 – 59 | 257 (10.1) | 228 (10.4) | 5 (5.4) | 8 (13.8) | 8 (10.4) | 8 (6.5) | 0 (0.0) |
| 60 – 64 | 240 (9.4) | 215 (9.8) | 9 (9.7) | 4 (6.9) | 6 (7.8) | 6 (4.9) | 0 (0.0) |
| 65 – 69 | 141 (5.5) | 129 (5.9) | 6 (6.5) | 3 (5.2) | 3 (3.9) | 1 (0.8) | 0 (0.0) |
| 70 – 74 | 72 (2.8) | 68 (3.1) | 1 (1.1) | 2 (3.4) | 0 (0.0) | 1 (0.8) | 0 (0.0) |
| >75 years | 54 (2.1) | 51 (2.3) | 1 (1.1) | 1 (1.7) | 0 (0.0) | 1 (1.7) | 0 (0.0) |

Note. a: We recognize that sex and gender differ, but were limited by the various concepts of these terms in the wide range of cultures in the over 130 countries in this study. Please see the limitations portion of the Discussion section for further details on this issue. b: The region where a given participant resides.

Table S3. Examples of supporting quotes for each theme and sub-theme identified in the survey data.

| Theme | Sub-theme | Quote |
| --- | --- | --- |
| Theme 1: Delays and restrictions | Changing COVID-19 regulations | “Occasionally, COVID-related considerations took priority, pushing academic ones back (participant number ID537, male, Turkey).”  “Most of the reduction in scientific productivity went into ever-changing administrative means, regulations, communications, etc. to adopt to new pandemic measures (ID1616, male, Austria).”  “Due to increased management and administrative activities to implement COVID-19 safety (and other) measures and COVID-19-related meetings, I have less time to be productive (ID789, male, USA).” |
|  | Lack of staff and support | “The university eliminated many positions so much of administrative work now falls on me (ID519, male, USA).”  “The financial stress of the university led to massive layoffs. Though our Dept was spared, resulting infrastructure changes (e.g., less pre-post award grant support, eliminated IT and admin support) meant that as a leader these routine duties fell to faculty. With research duties plus this impact, reduced productivity (ID2405, female, USA).”  “Difficulty recruiting new team members or students has led to decreased staffing (ID1900, male, Australia).”  “Difficulty recruiting new team members has significantly slowed research (ID2339, female, USA).” |
|  | Banned access to laboratories/office/travels and recruitment | <Ban on access to laboratories, offices, and work travel>  “As an experimental scientist and P.I., the limited accessibility of lab resources and recruitment of talented personnel reduced the scientific productivity significantly (ID266, male, USA).”  “The pandemic cancelled a lot of planned travels and affected the international contacts, amount of collected data, and arriving of expected new technologies for my research (ID1026, male, Bulgaria).”  “COVID-19 pandemic limited access to those data sources of interview, observation, and survey. Thus, results cannot be generated and the following processes after data gathering must be postponed or delayed (ID1739, male, Japan).”  <Delayed recruitment>  “Our major project essentially ground to a halt because of the inability to recruit subjects, inability to evaluate them in-person, and the reluctance/inability of subjects to come to the site (ID432).”  “Limitations on personnel on campus mean that I conducted experiments myself with less time for writing and more administrative tasks (ID977, male, USA).”  “The delayed postdoc visa from three different countries and still waiting to receive a response while keeping my immigration status in my current resident country is getting challenging (ID1782, female, Canada).” |
|  | Shortage of research equipment | “We were unable to obtain animals and some reagents for months. We still have purchasing delays and campus wide shortages of plasticware and personal protective equipment. We have had to halt some experiments because we do not have necessary materials and I am a year behind intended publication schedule (ID1115, female, Australia).”  “Lack of essential plasticware and reagents for cell culture and RTPCR (author: RTPCR = reverse transcription polymerase chain reaction) because of shipping delays and unavailability (ID2012, female, USA).” |
|  | Canceled grants and delays in publication | “Funding agencies have no funds to support research. I have written more than 15 grant with no success. In the past I have been very successful but with reduced funds and more people applying success rate is low. The government has abandoned basic science funding (ID1128, male, Australia).”  “I had one major grant cancel at the last minute because of COVID (ID209, male, USA).”  “I sense a delay in reviewer reports that might be related to Covid during the publishing process (ID1047, male, Germany).”  “The most noticeable effect was the time required to find reviewers in some journals, the review process was extended (ID1041, female, Finland).” |
| Theme 2: Changes in responsibilities | Teaching burdens and online supervision | <Difficulties in teaching and supervision>  “Online teaching was very labor intensive and required extensive preparation compared to classroom teaching. All aspects of work were altered and had to figure out how to do things as we went along (ID490, female, USA).”  “Professors had to organize new course timetables and online teaching with little or no help from administrative personnel and very poor guidelines (ID1235, male, Spain).”  “The transition to remote teaching was in my case abrupt, unsuspected and I received next to no support in the first phase of the remote classes on teaching tools, e-learning system in use, etc. (ID383, male, Poland)”  “I had to spend much more time transitioning my classes online and responding to students who tested positive, were quarantined, had family who died, etc. (ID2520, female, USA)”  <Inefficiency of online teaching>  “Online platform is not effective because of time differences among countries. For teaching, it consumes time to prepare teaching material for effective online class (ID2457, female, Thailand).”  “One of the classes I teach involves lab experiments, and this class has been severely disrupted two years in a row now (ID2152, female, USA).”  “In teaching activity, the pandemic affected by poor feedback from students, lack of hardware, software for doing the online teaching and by the problem caused by low quality of internet connection at work (ID2016, male, Romania).”  <Improvement of online teaching>  “Teaching efforts were increased by making a lot of new education material for on-line use in the 1st year of the pandemic (2020) but in the end, that turned out to be an improvement for the 2021 teaching semesters (ID2209, gender non-conforming, South Korea).”  “The transition to online learning required a massive re-structuring of all courses within our faculty. To account for this, we increased our administrative work exponentially and this obviously required extremely high levels of productivity (ID662, female, Canada).” |
|  | No or little time for research | “My time was consumed with additional mentoring, transition of courses to online environments, and additional emails/meetings/paperwork. I felt spread very thin and was unable to maintain my prior research productivity (ID1582, female, USA).”  “I have spent much time devising accommodations to teaching and research to enable these to continue in a safe manner, including restructuring research projects, teaching and evaluation formats, and scheduling times to stay in contact with lab personnel. This resulted in less time for research (ID646, male, USA).”  “Grant applications were delayed due to lack of time but also due to reduced contact with colleagues as we are all working at home (ID354, female, USA).” |
|  | Increased burden for child/eldercare and domestic work | <Increased childcare>  “The assumption that the women have to bear the vast majority of the family responsibilities pushed all the homework on me with limited support from anybody else. This brought unnecessary stress on me and drastically limited my ability to properly carry out my job (ID2294, female, Italy).”  “(The pandemic) significantly reduced my productivity. My spouse has continued to work outside the home throughout the pandemic, whilst I am now at home, in a crisis, trying to work, but also look after my two young children and homeschool them (ID75, female, New Zealand).”  “I have the more 'flexible' job, so I am the default parent that was in charge before we could enter our child into daycare and now they are in daycare, but hours of often cut short or they are closed due to staffing issues, so responsibility comes to me (ID1556, female, USA).”  “The lack of daycare and in-person homeschooling reduced the hours I could work and reduced the productivity of working at home (ID2311, male, USA).”  <Equal division of childcare>  “My husband and I were managing our dual careers, 3 meals a day, and daycare/school for pre-K, elementary and middle school children at home. We traded a lot of sleep for work time (ID652, female, USA).”  “The most demanding was the on-line learning of children, me (and my wife and both parents) had to partly substitute the teachers (ID1561, male, Czech Republic).”  <Increased family care>  “My close family member fell ill in April 2020 in another city, with no alternative during the lockdown, I had to shift for several months to take care. There was absolutely no support from my organization and online teaching continued making it more stressful. Research activities shut down (ID1990, female, India).”  “Home care for parent unavailable and very expensive. Not a good situation, my career has been halted and I am unfounded, unlike my female colleagues who have no family—they have flourished. (ID31, female, USA).”  “It has been affected to the extent of having to do household chores while completing academic tasks when at home (ID1405, female, Ecuador).”  “I lost my house cleaners, so I had to spend more time on housework, which took away from research time (ID283, female, USA).”  <Workload for single participants>  “As a single person working from home, I have been expected to ‘compensate’ work-hours/efforts for my colleagues with families (ID110, other sex, USA).”  “As a single post-doc with no children, I was often expected to take on extra tasks (ID375, female, Germany).” |
|  | Increased number of online meetings | “Increase in the number of virtual meetings, impacting the amount of time spent on other productive tasks (ID1409, female, UK).”  “Huge increase in number of meetings and emails leaving little time for work in between (ID2279, female, UK).”  “I spend an extremely large number of hours on online meetings, compensating of classes (because there are a large number of absences from F2F exercises, etc.) (ID731, female, Croatia).” |
| Theme 3: deteriorated health | Deterioration of physical health | “I was not able to be as effective in manuscript writing, I was always behind on deadlines for grant submissions and administrative tasks. This was mostly due to the fatigue of trying to manage my home life and work life in the same space (ID1631, female, USA).”  “Decreased overall work productivity, I lost days when I was sick. I couldn't keep up with emails, notes, etc. I'm still not fully caught up months later (ID773, female, USA).”  “Less productive at home. Working at night make sleeping not easy (ID263, male, Italy).” |
|  | Deterioration of mental health | “I added an extra (unfunded) COVID-19 research projects so my productivity is great, but I am completely burned out (ID2153, female, USA).”  “Blurred the lines between work and home life as work moved to completely at home. Productivity increased, but burnout also increased (ID875, male, USA)”  “I live alone, and the isolation and loneliness made it very hard for me to concentrate on my work for the past 1 year (ID1717, male, USA)”  “I think the biggest challenge, as is true for many, is the stress and mental health difficulties that arise from being isolated (ID645, female, USA).”  “Overall productivity decreased significantly due to being isolated from lab and also due to the general stress and anxiety due to the pandemic (ID1658, male, USA).”  “The pandemic negatively affected my research mind. My motivation for research decreased at the pandemic probably because of the anxiety regarding health of me and my family (ID1957, male, Japan).”  “Overall, the lack of job security and increased workload has led to increased anxiety and loss of motivation (ID18, female, Bangladesh).” |
| Theme 4: Insufficient at-home work environment | Blurred boundary between work and life | “Working from home has blurred the lines of work/life balance and made it difficult to turn my mind off of work (ID844, female, USA).”  “Since I work at home, my superiors expect me to be always available, and I end up having trouble keeping regular working hours. I feel tired often (ID1159, female, Bulgaria).”  “Unspoken expectations of 'being available' all sorts of hours 'because I am right there anyway'. This has led to early starts and working into evenings plus increased administrative work around COVID-19 (ID1501, female, Canada)”.  “Increased productivity as colleagues and collaborators are more available online (ID526, male, Switzerland).” |
|  | Insufficient workspace and/or set-up | “Working from home without having a proper workspace resulted in inefficiency (ID2448, female, Canada).”  “Lower Internet speed led to less data processed (ID343, female, USA).”  “Since my work is largely computer modeling, I could do it from home. There were delays due to poor internet connection (ID2252, male, Germany).”  “The COVID-19 pandemic requires remote teaching/or work from home, which is not very effective considering the poor network or internet facilities (ID364, female, Indonesia).” |
|  | Lack of in-person interactions | “E-mail-based supervision is time consuming (ID922, male, Estonia).”  “As the Chair the administrative, work multiplied with most communications being by email or remote, problems that in person will get solved quickly drag for longer in email (ID1355, female, USA).”  “Lack of in person discussion and free chat, which often leads to interesting ideas and solutions to problems, and sometimes accidentally expands the research scope (ID1510, male, Switzerland)”.  “Lack of exchange with colleagues and difficulties in establishing new bonds and nurturing existing relations (affected productivity) as an early career scientist (ID558, female, Germany).” |
| Theme 5: Increased flexibility | Teaching environment | “I have more flexibility in teaching and teaching methods as I don't need to book lecture halls and can experiment with different techniques (ID561, male, Sweden).”  “Remote teaching of programming and project courses worked better than in-person as the students were working with computers. Zoom breakout room was great for project-based courses (ID1399, male, USA).” |
|  | Grants for COVID-19 research | “Actually (the pandemic) increased scholarly activities as they expanded to include SARS-CoV-2 and COVID work. They are directly related to our clinical and public health research interests (ID1157, female, Canada).”  “I had the opportunity to work on a COVID-19 related project that was launched in March 2020 in my lab. This is an international project with funding from high-profile organizations. Valuable research experience, looks good on my CV (ID949, female, France).”  “I wanted to understand the reasons of the COVID-19 pandemic; therefore, I had been working on this subject for about one year (ID2377, male, Hungary).” |
|  | Alternative methods | “Due to lab restrictions, I had plenty of time to check old data that were waiting for analysis and publication. Consequently, my publication record increased during the COVID-19 period, considerably (ID1834, male, Norway).”  “Bench lab productivity greatly decreased. This was adjusted by increased writing and getting papers out for both me and students (ID1970, male, USA).”  “Due to the lack of experimental studies my work has primarily focused on surveys and other types of research that does not require direct contact with participants (ID1562, male, Netherlands).”  “I have been able to continue publishing only by working on review papers and survey-based studies (ID293, male, Canada).”  “We have paradoxically seen an increase in research output in terms of papers, but we have now reduced our new data coming in that will impact on future productivity (ID1936, male, Australia).”  “Some aspects improved, such as getting papers published, but overall, it decreased my ability to collect data and so will negatively affect long-term publishing and moving my research agenda forward (ID897, male, USA).”  “At the beginning I had more time to write the manuscripts but then it was really difficult to collaborate and I lost a lot of data and experiments and this will lead to a slowdown in publications in the next future (ID2352, male, Italy).” |
|  | Fluidity of workday | “It increased my overall work productivity when I was working remotely at home 100% of the time because I am more productive when working at home. There are fewer distractions, I am more outcomes-oriented, and can focus at my optimum times for working when working remotely (ID975, female, USA).”  “Teaching online and not commuting to campus increased the time I had available for research (ID988, female, USA).”  “Not travelling to/from work and avoiding transitions around campus for meetings allowed more time to focus on grants, writing, establishing a better work-life balance, and improving mental health (ID966, female, USA).”  “The COVID-19 pandemic has increased work productivity. It has saved time from transportation; provided flexibility for interaction through internet platforms at my convenience; enabled watching a number of webinars and interact with various communities that would not be possible through face-to-face interaction (ID87, female, Greece).” |

Table S4. The number of participants who reported each of the found themes in this research by geographical regions (*n*=2548).

| Region | Theme 1 | Theme 2 | Theme 3 | Theme 4 | Theme 5 |
| --- | --- | --- | --- | --- | --- |
| Africa | 41 (44.1%) | 13 (14.0%) | 15 (16.1%) | 14 (15.1%) | 14 (15.1%) |
| Asia | 148 (34.7%) | 80 (18.8%) | 54 (12.7%) | 82 (19.2%) | 53 (12.4%) |
| Caribbean | 3 (60.0%) | 3 (60.0%) | 1 (20.0%) | 1 (20.0%) | 0 (0.0%) |
| Europe | 232 (26.9%) | 195 (22.6%) | 142 (16.5%) | 198 (22.9%) | 193 (22.4%) |
| North America | 337 (39.9%) | 216 (25.6%) | 125 (14.8%) | 156 (18.5%) | 191 (22.6%) |
| South America | 63 (40.4%) | 40 (25.6%) | 32 (20.5%) | 25 (16.0%) | 25 (16.0%) |
| Oceania | 62 (47.0%) | 42 (31.8%) | 27 (20.5%) | 24 (18.2%) | 21 (15.9%) |
| Other | 1 (16.7%) | 1 (16.7%) | 0 (0.0%) | 1(16.7%) | 1 (16.7%) |
| Total | 892 (35.0%) | 593 (23.3%) | 402 (15.8%) | 506 (19.9%) | 502 (19.7%) |

Note. The percentages in the table indicate the portion of the participants among all participants from each geographical region.


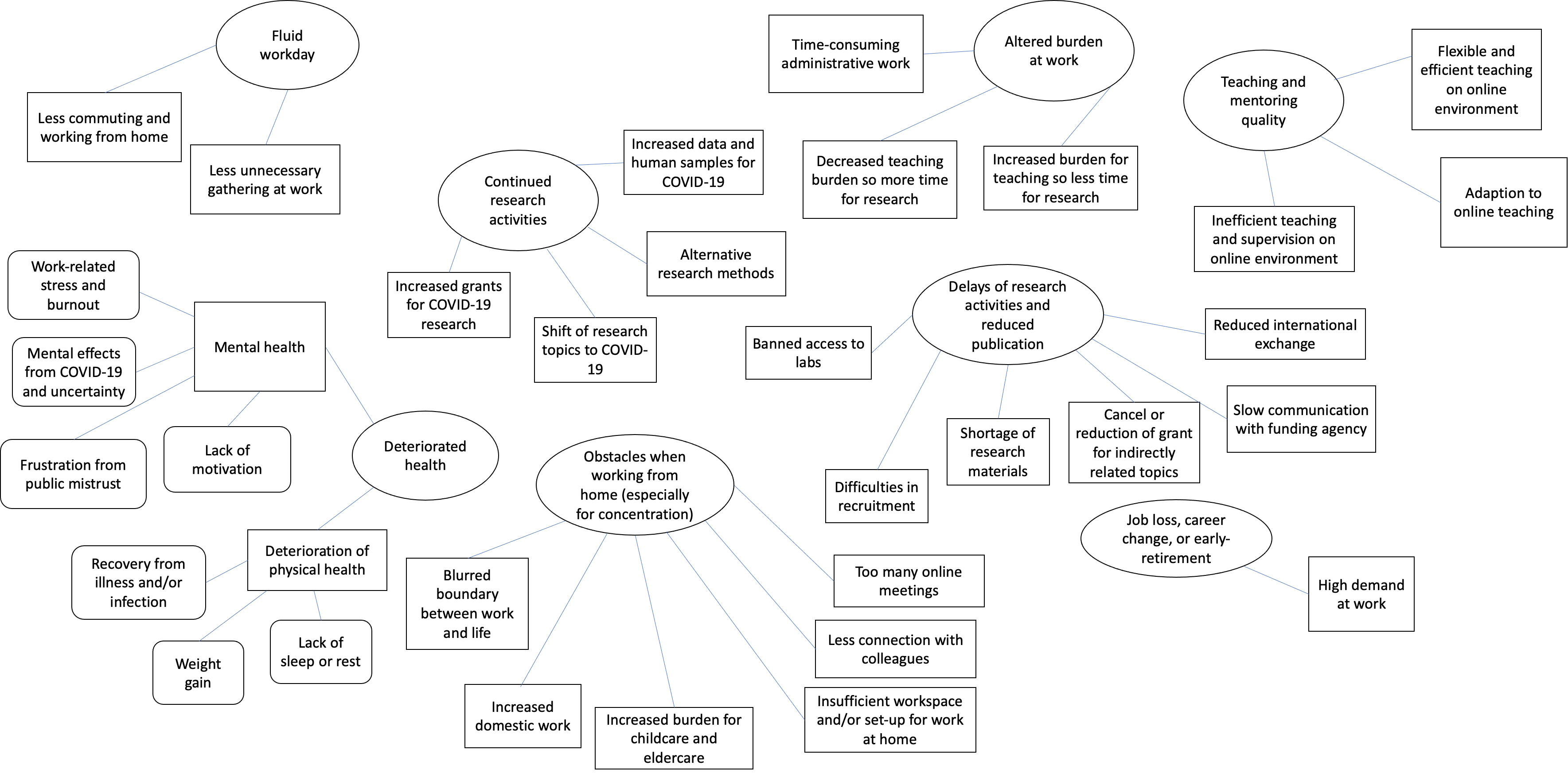


Figure S1. Initial thematic map, showing 8 potential main themes - Version 1.

*Note*. Circles represent themes and squares represent sub-themes.


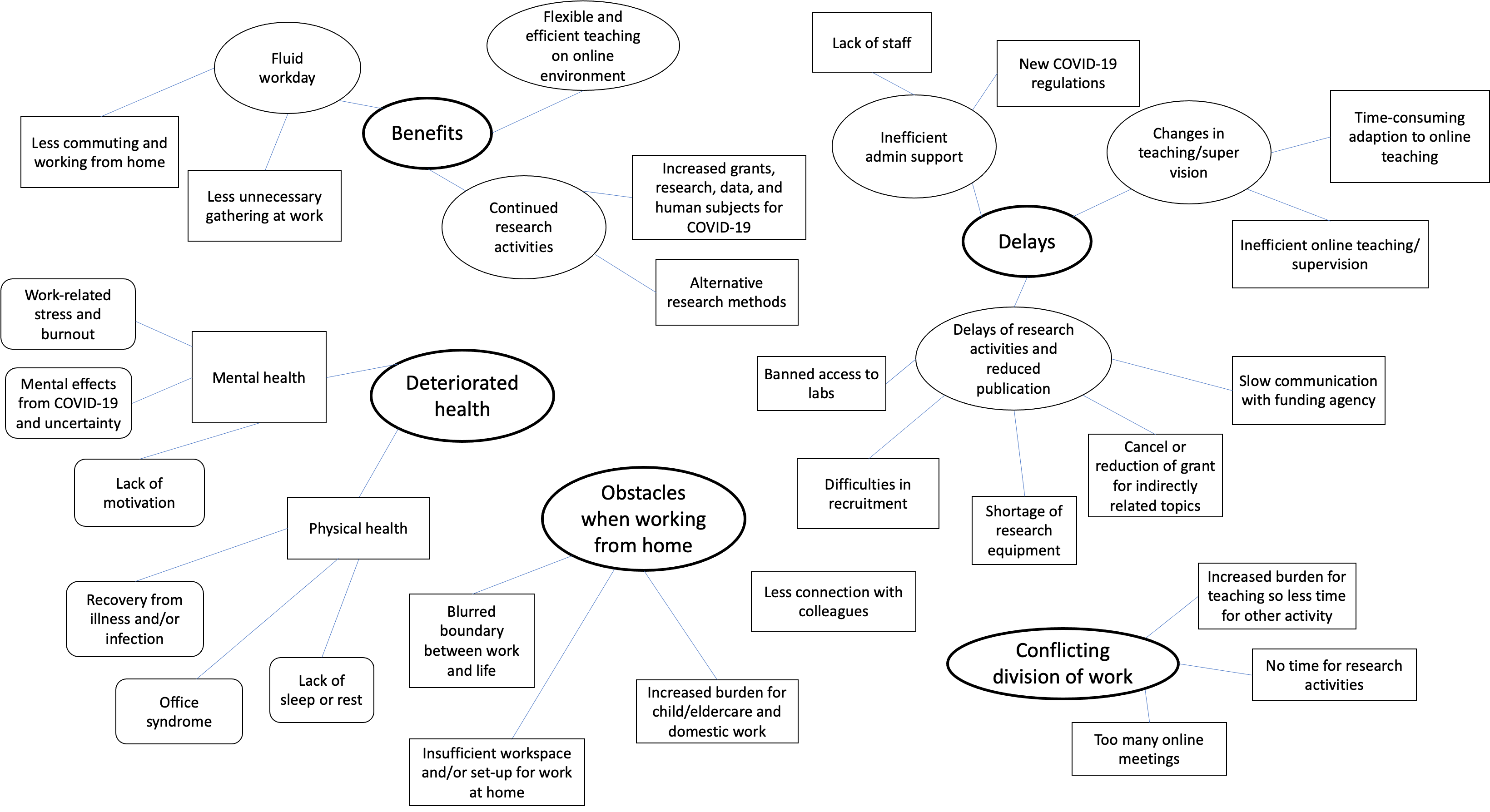
Figure S2. Version 2 thematic map, showing 5 potential main themes.

*Note*. Circles with a thick border are the new higher themes grouping the themes identified in version 1 in Figure S1.

Table S5. Research questions of previous studies and the current study.

| Research questions | Study | Study region/participants |
| --- | --- | --- |
| - The impact of COVID-19 on scientists’ perception of scientific and academic productivity - Inequitable changes in duties and responsibilities for caregiving at home by gender and status as a parent | This research | 2,548 participants from 132 countries |
|  | (Yildirim and Eslen‐Ziya 2021) | 460 participants from the US and Europe |
|  | (Górska et al. 2021) | 28 participants from Poland |
| - The impact of COVID-19 on scientists’ perception of research productivity | (Krukowski et al. 2021) | 284 participants from the US |
|  | (Breuning et al. 2021) | 1,003 participants from various regions |
| - The impact of the pandemic on perception of productivity of female scientists | (Walters et al. 2022) | 2,029 women from South Africa |
|  | (Bender et al. 2022) | 51 women from the US, Europe, and Africa |
|  | (Minello et al. 2021) | 50 women in Italy |
| - Overall (positive and negative) experience of scientists during the pandemic regardless of gender | (Sarah et al. 2021) | 210 respondents from various countries |
| - Changes in the number of scientific work hours during the pandemic by gender | (Deryugina et al. 2021) | 27,991 participants from various countries |
|  | (Myers et al. 2020) | 4,535 participants from the US and Europe |
| - Experience of teaching under the COVID-19 lockdown among university teachers | (Kovacs et al. 2022) | 10 participants from Europe |
|  | (Abdelmola et al. 2021) | 163 participants form Saudi Arabia |
| - Impact of COVID-19 on research publications by gender | (Abramo et al. 2022) | Publication database records |
|  | (Staniscuaski et al. 2021) | 3,345 Brazilian academics |
|  | (King and Frederickson 2021) | Publication database records |
|  | (Brown et al. 2021) | Publication database records |
|  | (Pinho-Gomes et al. 2020) | Publication database records |
| - Gender impact of COVID-19 policies at universities | (Sutherland et al. 2021) | Australia |
|  | (Nash and Churchill 2020) | Australia |

**References**

Abdelmola AO, Makeen A, Hanafi HM, Ageeli E (2021) E-learning during COVID-19 pandemic, faculty Perceptions, challenges, and recommendations. MedEdPublish 10:

Abramo G, D’Angelo CA, Mele I (2022) Impact of Covid-19 on research output by gender across countries. Scientometrics 1–16. https://doi.org/10.1007/s11192-021-04245-x

Bender S, Brown KS, Hensley Kasitz DL, Vega O (2022) Academic women and their children: Parenting during COVID‐19 and the impact on scholarly productivity. Fam Relat 71:46–67

Breuning M, Fattore C, Ramos J, Scalera J (2021) The Great Equalizer? Gender, Parenting, and Scholarly Productivity During the Global Pandemic. PS Polit Sci Polit 54:427–431. https://doi.org/10.1017/S1049096520002036

Brown C, Novick TK, Jacobs EA (2021) Gender Disparities in Authorship of Invited Manuscripts During the COVID-19 Pandemic. Womens Health Rep 2:149–153. https://doi.org/10.1089/whr.2021.0023

Deryugina T, Shurchkov O, Stearns J (2021) Covid-19 disruptions disproportionately affect female academics. AEA Pap Proc 111:164–168. https://doi.org/10.3386/w28360

Górska AM, Kulicka K, Staniszewska Z, Dobija D (2021) Deepening inequalities: What did COVID‐19 reveal about the gendered nature of academic work? Gend Work Organ 28:1546–1561. https://doi.org/10.1111/gwao.12696

King MM, Frederickson ME (2021) The Pandemic Penalty: The Gendered Effects of COVID-19 on Scientific Productivity. Socius 7:1–24. https://doi.org/10.1177/23780231211006977

Kovacs H, Zufferey JD, Tormey R, Jermann P (2022) Teaching under lockdown: the change in the social practice of teaching. High Educ 1–19. https://doi.org/10.1007/s10734-022-00863-3

Krukowski RA, Jagsi R, Cardel MI (2021) Academic Productivity Differences by Gender and Child Age in Science, Technology, Engineering, Mathematics, and Medicine Faculty During the COVID-19 Pandemic. J Womens Health 30:341–347. https://doi.org/10.1089/jwh.2020.8710

Minello A, Martucci S, Manzo LK (2021) The pandemic and the academic mothers: present hardships and future perspectives. Eur Soc 23:S82–S94. https://doi.org/10.1080/14616696.2020.1809690

Myers KR, Tham WY, Yin Y, et al (2020) Unequal effects of the COVID-19 pandemic on scientists. Nat Hum Behav 4:880–883. https://doi.org/10.1038/s41562-020-0921-y

Nash M, Churchill B (2020) Caring during COVID‐19: A gendered analysis of Australian university responses to managing remote working and caring responsibilities. Gend Work Organ 27:833–846

Pinho-Gomes A-C, Peters S, Thompson K, et al (2020) Where are the women? Gender inequalities in COVID-19 research authorship. BMJ Glob Health 5:e002922. https://doi.org/10.1136/bmjgh-2020-002922

Sarah K, Oceane S, Emily F, Carole F (2021) Learning from lockdown-Assessing the positive and negative experiences, and coping strategies of researchers during the COVID-19 pandemic. Appl Anim Behav Sci 236:105269. https://doi.org/10.1016/j.applanim.2021.105269

Staniscuaski F, Kmetzsch L, Soletti RC, et al (2021) Gender, race and parenthood impact academic productivity during the COVID-19 pandemic: from survey to action. Front Psychol 12:1–14. https://doi.org/10.3389/fpsyg.2021.663252

Sutherland G, Vazquez Corona M, Bohren M, et al (2021) A rapid gender impact assessment of Australian university responses to COVID-19. High Educ Res Dev 1–15

Walters C, Mehl GG, Piraino P, et al (2022) The impact of the pandemic-enforced lockdown on the scholarly productivity of women academics in South Africa. Res Policy 51:104403. https://doi.org/10.1016/j.respol.2021.104403

Yildirim TM, Eslen‐Ziya H (2021) The differential impact of COVID‐19 on the work conditions of women and men academics during the lockdown. Gend Work Organ 28:243–249. https://doi.org/10.1111/gwao.12529
